# The effectiveness of using in-game cards as reward

**DOI:** 10.1186/s41039-017-0054-8

**Published:** 2017-08-01

**Authors:** Peayton Chen, Rita Kuo, Maiga Chang, Jia-Sheng Heh

**Affiliations:** 1 Department of Information and Computer Engineering, Chung Yuan Christian University, Taoyuan, Taiwan; 2 Department of Computer Science and Engineering, New Mexico Institute of Mining and Technology, Socorro, USA; 3 School of Computer and Information Systems, Athabasca University, Athabasca, Canada

**Keywords:** Trading card game, Elementary school, English learning, Educational reward, Learning motivation, Competition, Game

## Abstract

The research team has developed a web-based multiplayer trading card game to allow teachers choosing cards as rewards for students who actively participate in discussions and classroom activities as well as perform well in terms of doing assignments and writing exams or quizzes. In order to verify the effectiveness of the use of in-game cards as rewards, the research team integrated the trading card game into a web-based English vocabulary learning system. Students can receive cards as rewards every time after they use the learning system. A 6-week experiment had been conducted at an elementary school with 172 fifth-grade students. The results showed that boys have higher intention of getting the in-game cards as rewards. The research also showed that the use of the in-game cards as educational rewards not only motivates students to use the vocabulary learning system but also improves their learning outcome. The research result supported the recommended process for teachers to adopt the trading card game in their courses.

## Introduction

Past research shows that symbolic educational rewards sometimes are not so meaningful to students (Kohn 1999). On the other hand, students would not appreciate if the real educational rewards are not so useful to them (Wu and Elliott 2008). Of course, “money” as rewards should not be considered (McNinch 1997). Although researchers have different opinions about the effect of rewards, rewards do provide short-term incentive immediately to intrinsic motivation as well as learning performance (Winefield et al. 1984; Witzel and Mercer 2003). Card games are very common and welcome by students in different ages and even for graduate and undergraduate students. But most of the card games are either commercial ones or difficult to take as educational reward systems due to its in-game elements are hard to connect to the academic performance of learning activities.

The research team has developed a discipline independent trading card game in which the in-game cards can be delivered as rewards by any teacher in any course. Trading card game (TCG) is also called collectible card game or customizable card game. After the first TCG, Magic: The Gathering, was introduced in 1993, Pokémon and other TCGs have been developed and have successful market hit (David-Marshall et al. 2010). Unlike other card games, such as the poker or UNO, TCGs have more cards than the poker and UNO: poker has only 56 cards and UNO has 108 cards, whereas TCGs always have hundreds of cards. Moreover, cards in TCG are extendable, which means, new cards can always be designed and provided when the game needs. This feature is extremely important when we want to use in-game cards as reward since we do not want to see students losing their interest just because they have seen all of the cards and have a full house collection. An open-access trading card game^1^ was developed by the research team intended for teachers delivering or giving cards to students according to their performances in different learning activities (e.g., classroom participation, discussions, assignments, quiz, exams).

The “Related works” section starts with the introduction of how the game is integrated in learning and the role of rewards in game-based learning. The design of the trading card game is described in the “Trading card game” section. The “Methods/experimental” section proposed several hypotheses to the effect of the trading card game and provided details of the experiment design for verifying the hypotheses. The collected data is analyzed and discussed in the “Results and discussion” section. The “Findings and recommendations” section lists the findings of the experiment and proposes some recommendations for teachers who would like to adopt the trading card game in their courses. Conclusion is made in the “Conclusion” section with further works the research team plans to do for next.

## Related works

### Game-based learning

Digital game industry becomes the mainstream of the market (Siwek 2014; Rideout et al. 2010) because fantasy, curiosity, challenge, and control are the features that attract players (Malone and Lepper 1987). Some researchers point out that playing can hold student attentions and make learning be more interesting (Virvou, Katsionis, & Manos, 2005; Boyle 1997). In fact, games can produce engagement and delight in learning (Boyle 1997). For these reasons, many studies use commercial games directly or design new education games and have evidences of students can get significant improvement in learning.

Games can be classified into different genres, such as simulation, strategy, and role-playing (Apperley 2006). Simulation game simulates the dynamics of the system in the real world and help learner learn practical skills by understanding how decisions may cause the results in the simulated environment (Dankbaar et al. 2016). Take Shakshouka Restaurant game in the My Money website as an example; the game helps students develop skills in financial and math (Barzilai and Blau 2014).

Strategy games are the most popular genre in the education game (Hainey et al. 2016) because the players is using the god’s-eye-view to see the virtual world (Apperley 2006). Take Maguth and colleagues’ study for example; they used Age of Empires II: The Age of Kings to teach seventh-grade students in a social study class (Maguth et al. 2015). In the game, students can understand what happened when two cultures encounter in the Middle Ages. The narrative in role-playing game can help students develop the disciplinary knowledge when experiencing through the story (Cheville 2016). Chang and colleagues integrated the challenge, control, and fantasy game features into a mobile game for museum learning (Chang et al. 2014). Students can role play as a cinema property handler or an artist in the game and find the museum’s artifact that its properties, such as dynasty or materials, fit the requirements their roles’ clients in the game ask for.

Trading card game has also been used as tools to enhance learning. In teaching host defense concept, Steinman and Blastos (2002) have designed a board game to teach the principles of host defense in biomedical lesson. Students have to understand the concepts of immunology and infectious disease in order to use the correct pathogen cards to attack opponents’ organs or defense their own organs. Another educational application of TCG is the Weatherlings, an online collectible card battle game developed by MIT’s Scheller Teacher Education Program to teach weather and climate (Klopfer et al. 2012; Sheldon et al. 2010). In Weatherlings, players have to predict the weather in the match according to the previous climate data of the arena; they have to choose proper cards which fit the climate to make more damage to their opponents.

### Rewards in game-based learning

In digital games, reward systems can keep players’ interests in playing the games (Hallford and Hallford 2001). Wang and Sun (2011) believe that the digital in-game rewards can be effectively motivating tools for educational purpose. In traditional learning or e-learning, after the students finished a learning activity or written an exam, they usually receive feedback from the teacher or the system. The feedback may be a summary of the learning activity they just finished, for example, a total score presenting their performance, a brief comment to suggest them how to do better, and/or some information for them what to learn further. Reward is also one of the feedbacks.

In Chang and colleagues’ research, they provided jewels as reward when learner accomplishes one quest in the mobile game in the museum (Chang et al. 2008). Wu and Elliott (2008) indicated three types of rewards shown in Table 1. They found that different students have different preferences toward the rewards. Gifted students preferred competition rewards, whereas non-gifted students preferred chance rewards (Wu and Elliott 2008). Rewards can be given not only by teachers but also by students. Pedro et al. (2015) designed a badging system which all participants, including teachers and students, in the educational web platform can design badges as rewards for other participants (Pedro et al. 2015). For example, a student can give badges to other students in the same group when he or she believes that the other students have good involvement in the activity.

**Table 1:** Reward types

Type	Description
Competition reward	The rewards are limited and must to compete to get.
Performance reward	Get the rewards according to the performance improvement.
Chance reward	Can get according to chance and do not need to pay any effort.

## Trading card game

### In-game cards as rewards

The proposed game is a discipline independent trading card game in which the in-game items can be delivered by any teacher as rewards in any course, grade, and school level. The in-game items are cards that students need to use while playing with others. Interpersonal motivation is important in learning (Malone 1981). Malone and Lepper propose that competition is an approach to create interpersonal motivation (Schwabe and Göth 2005; Malone and Lepper 1987). This research uses the players’ scores and ranks in the game to establish a competitive environment. By depleting opponent’s cards, students can earn scores for rank promotion. The ranks can intrinsically motivate students.

Well-designed peer competitions have been proved as a good way to get students motivated, and it is the basic idea of the game. In order to make students have the correct perception and positive attitude toward the competitions, a student’s ranking among all students is based on her or his scores rather than how many matches she or he has won or lost before. The student can get scores for the efforts she or he has done in the match, so she or he can still receive scores even she or he loses the match; sometimes, a student who loses a match could even receive more scores than the winner of that match because she or he has tried hard and never gave it up.

A student might be able to have more options and strategies in the match if she or he has more cards and even might be able to defeat his or her opponents easier. The only way he or she can get new cards including higher-level or rarer cards is to have good enough performance on the learning activities the teachers designated.

For those students who do not want to compete with others, the in-game items (i.e., the cards) have collectible features just like coins, stamps, hockey, and baseball cards; students may want to see higher-level cards as well as rarer cards in their collection. The effects of the in-game cards as rewards will be kept in student’s mind, and the learning motivation engaged by such mechanism may be carried to the followed courses, grades, or school levels. The students may want to get better in-game cards in the followed courses by learning harder and putting more efforts in the assignments, participation, discussions, and etc.

### Game features

More details of the game design can be found in Chen et al. (2009). In this paper, only the features and the game-play are discussed. The trading card game has six features:

- It is an online multiplayer game. If a game provides poor game experience, no one wants to play it. To avoid that, the first issue of this research is to make the game interesting. Competition and comparability can make games more interesting. Since the game needs to have at least two players to fight each other, it satisfies basic competition and comparability. For the improvement, we design the game as an online multiplayer game; thus, players can play together without physical limitation; for example, a student can stay at home and play with his/her fellows. Besides, we also design “audience mode”; thus, students can not only have matches with others but also be an audience of other matches. By watching others’ matches, they can learn the game-play strategies from others. Every match is recorded so they can watch replays at anytime, anywhere. As long as the students are interested in playing the game, they will be motivated to learn and actively participate and put more efforts on the activities their teachers designed in order to get more powerful and rarer cards.
- It can be played at many platforms. The game client is developed with Adobe Flash, and it can be played on any platform which has Flash player supports. So far, the game has been tested on Windows desktop and laptop computers as well as Android smartphones and tablets.
- It is a subject independent game. The in-game cards can be collected from different courses if the teachers adopt the game; therefore, students can collect cards from any courses via working actively and hard on the learning activities.
- The in-game cards can be delivered automatically and adaptively. Students shall be able to receive cards from time to time for their performance on learning activities in classroom, out of classroom, and after school. Teachers’ workload will increase if they are asked to deliver the cards manually on their own. The game provides server-side scripts which allow developers to design module and plug-in for existing learning management systems (e.g., Moodle) so the cards can be delivered to a student automatically when the student’s assignment being marked or the student’s participation in a discussion forum reach the awarding criteria pre-defined by the teacher.
- It is a multilingual game. The game supports multiple languages such as English and Chinese. The multilingual feature allows students from different countries playing with each other without language barrier. It only takes one day to add the support of a new language.
- The game client automatically checks for the new version and reminds the students to download new version of the game. From time to time, the game client may need to update as new gaming functions may be developed and added into the game. The game will check whether or not a new version is released when the students sign themselves in. If a newer version exists, the game will ask the students to download the new version for playing.

### The game

**Fig. 1:**
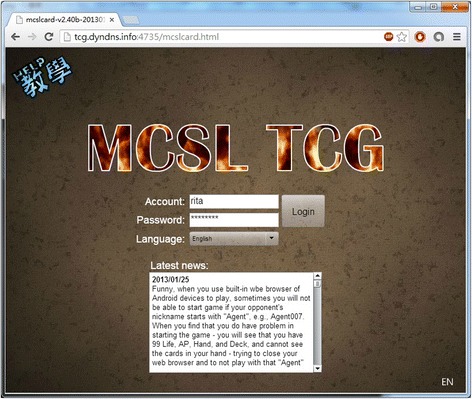
The trading card game

The research team chooses to use the Adobe Flash to implement the game client and uses open-source Project Darkstar to implement the game server. We also use JavaScript Object Notation as the data exchange format. Figure 1 shows the sign-in user interface of the game. Students can sign-in with the account and the password self-registered or received from their teachers. On the screen, the students can also see the latest news of the game (and the class) and access the tutorial of game-play (i.e., the HELP icon at the top-left corner).

**Fig. 2:**
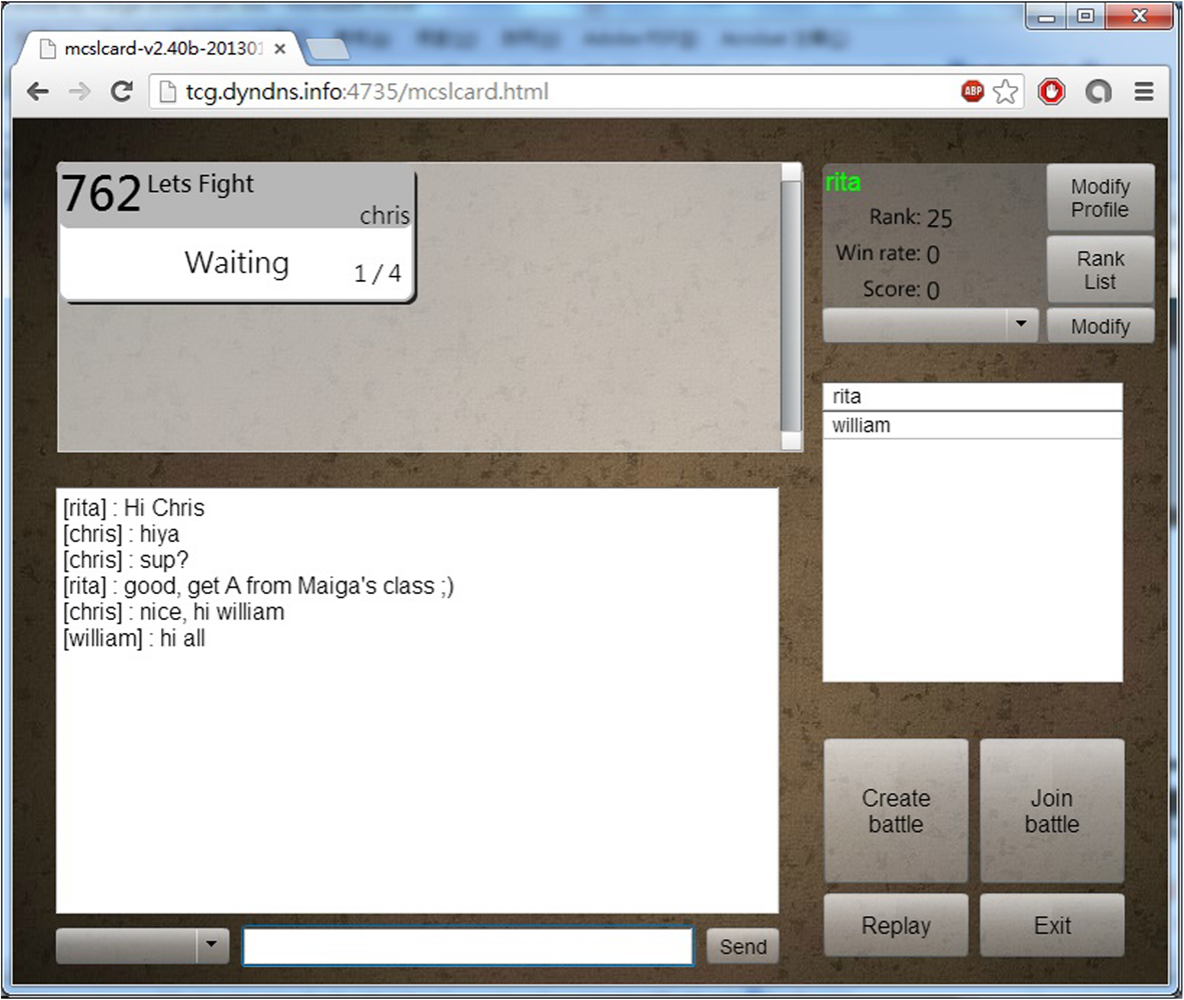
The game lobby

**Fig. 3:**
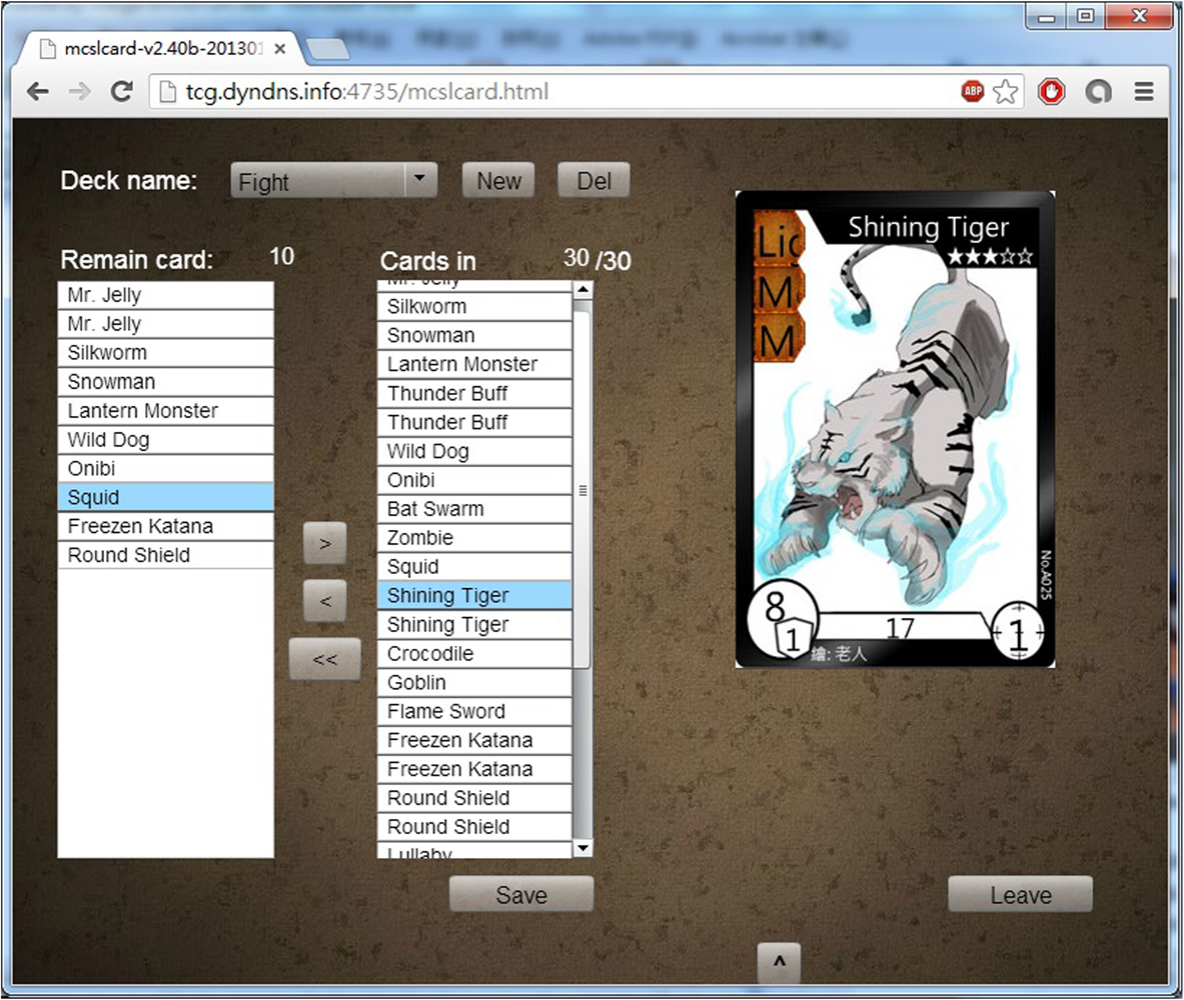
Decks editing room

After students sign-in the game, they are at the game lobby as Fig. 2 shows. The top-left panel shows existing matches happened in different rooms, waiting or battle room, in which maximum four players (i.e., two opponents and two audiences) are playing. The bottom-left chat panel allows players to communicate with others. The top-right personal information panel shows players’ information including their rank, winning ratio, and accumulated game score. Players can also create and (re-)arrange their decks for different gaming strategies they may want to use while playing with different players. The middle-right player list panel shows the players who are not in a match and hanging out at the game lobby. Four buttons include the creation of a battle room for match, join an existing battle room, replay existing game-play recordings, and exit from the game and can be found at the bottom-right corner on the screen. Figure 3 shows the deck editing room. Players can create, modify, and delete decks in this room. Once players have their decks ready, they can create a waiting room or join an existing waiting/battle room for having a match with others.

**Fig. 4:**
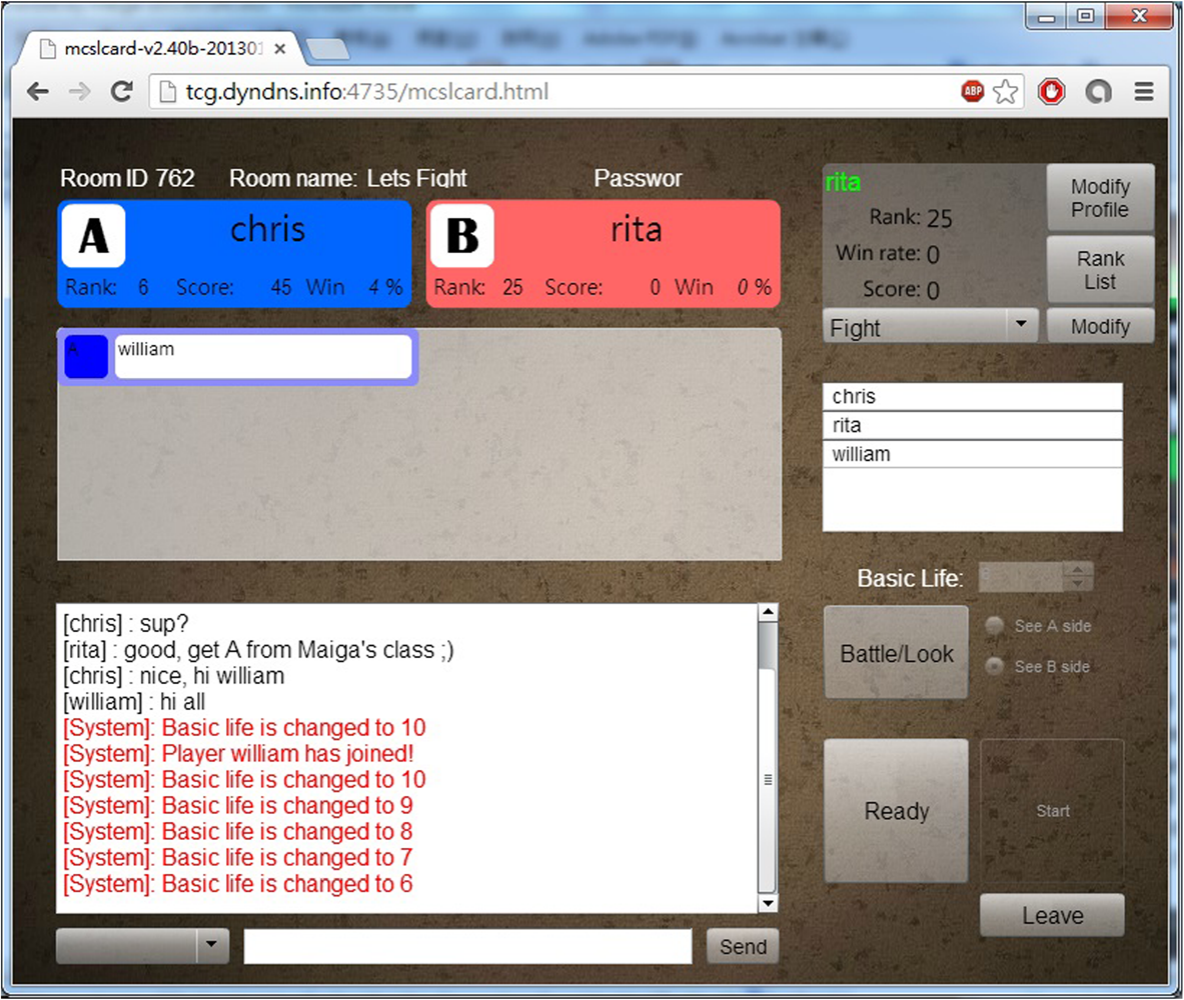
Waiting room

Figure 4 shows the waiting room. Players can wait for their opponent as well as audiences here. The player who creates the room for a match can setup the basic life points before the match starts. Less basic life points implies less time a match may take. Usually, a game may take up to 15 min when the basic life points are set to 5. Players can also choose whether they want to be the audience or the fighter of the match here. Two large square buttons at the bottom-right corner on the screen are used by fighters to tell their opponents they are “Ready” for the match. Only when all fighters in the room indicate they are ready, the “Play” button is enabled and the match starts.

**Fig. 5:**
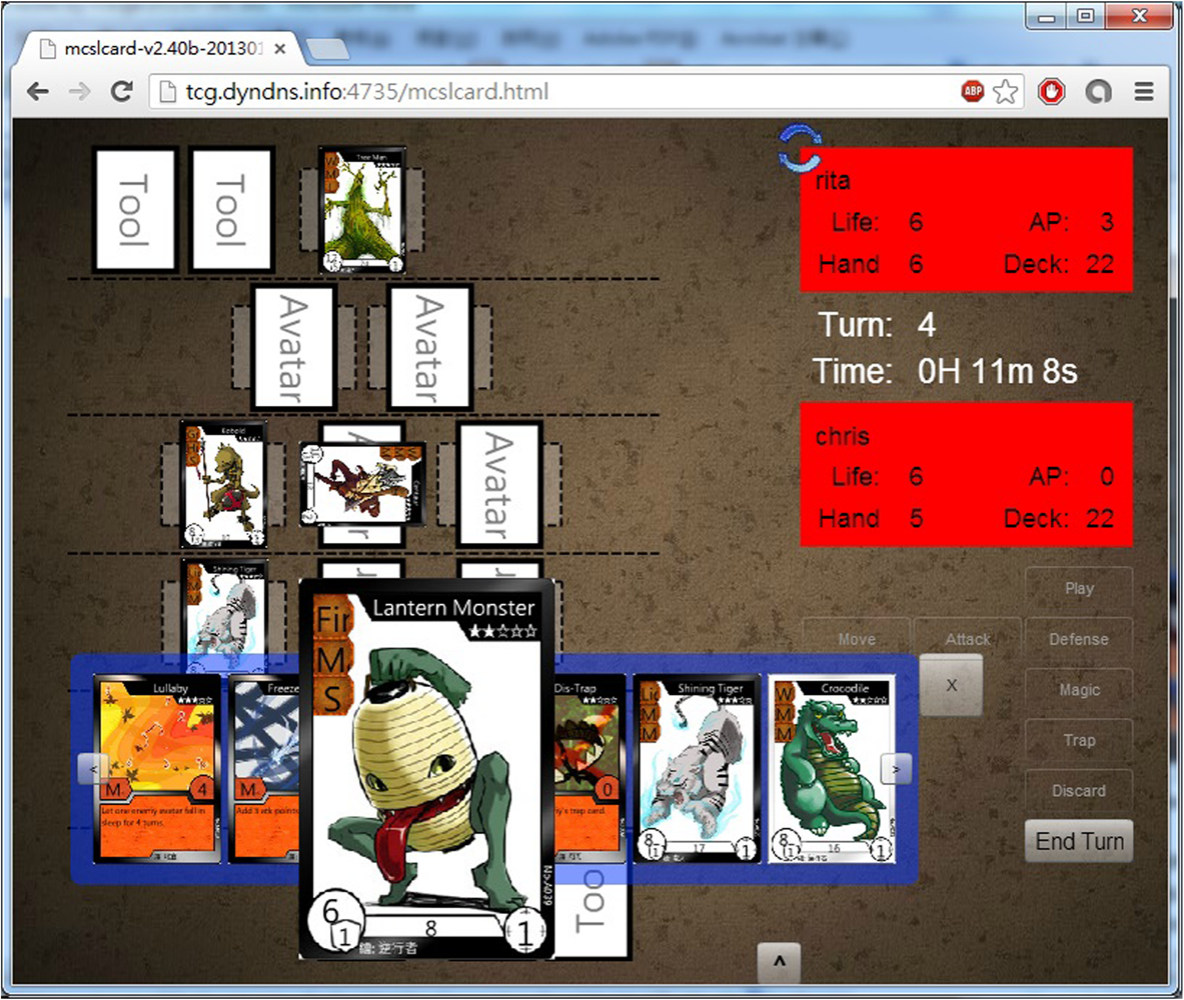
The screen of a match

**Fig. 6:**
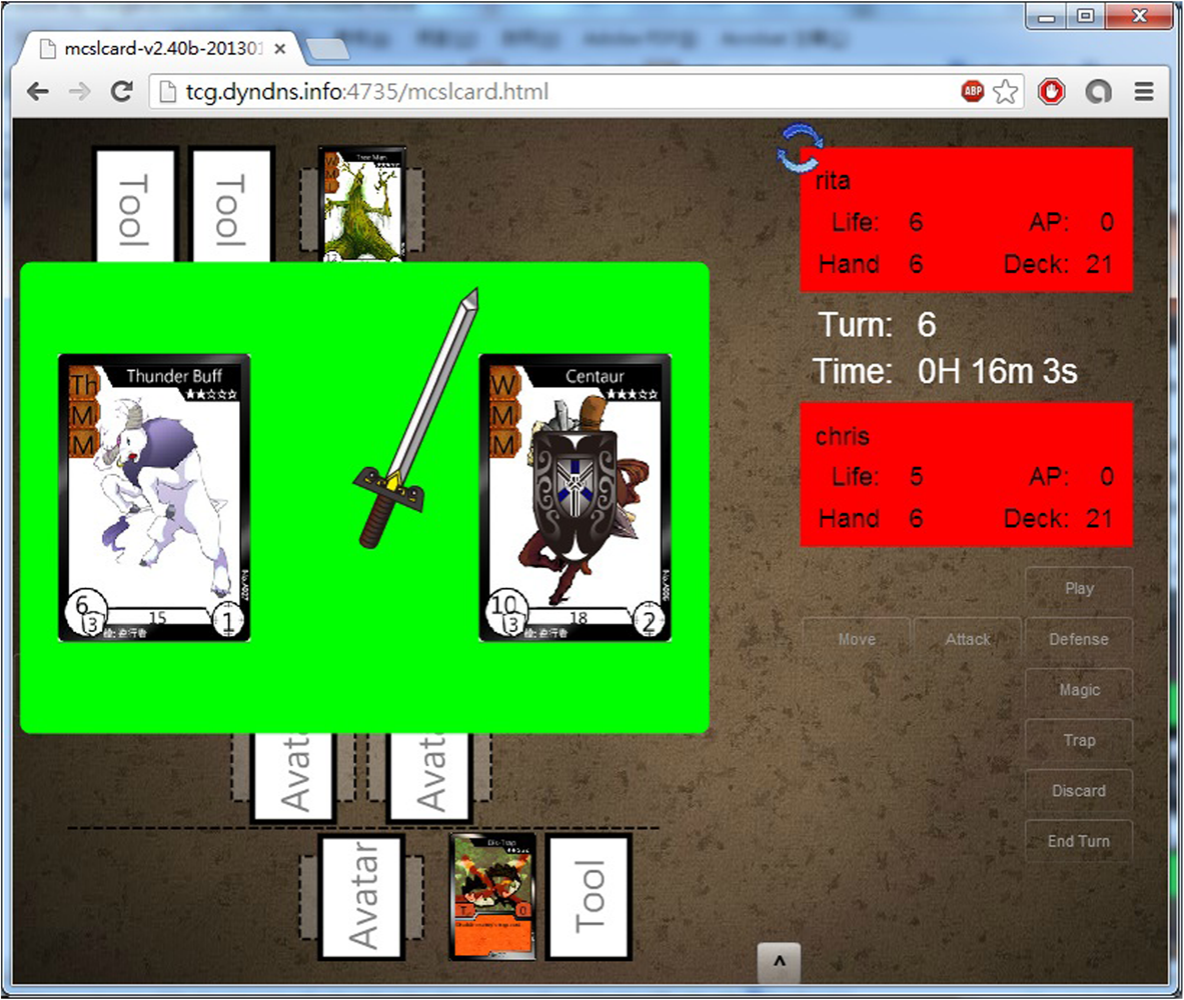
Combat animation

**Fig. 7:**
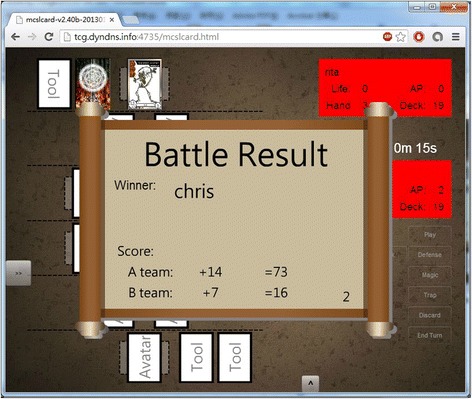
Match result

Figure 5 shows the screen of a match. It contains the battle field where the fighters can put their card on and fight with the cards their opponents put and the hand field where they can find what cards they have drawn from the deck; the information panel which shows fighters’ stats and turns; and a set of buttons for the game-play that players can use. Figure 6 shows the animation while a fighter chose to use his/her avatar to attack another avatar his or her opponent has on the battle field, and Fig. 7 shows the result of a match.

## Methods/experimental

### Experiment settings

To keep students’ motivation of putting efforts on doing learning activities, this research offers the game for students to use the cards they received from the use of a web-based English vocabulary learning system. The game not only increases the usability of the rewards but also stimulates students’ learning motivations because the more the vocabulary they learned or the better they performed in the quizzes, the more and better cards they can receive for playing the game and the higher the opportunity they have to defeat their fellows. The English vocabulary learning system gives students appropriate cards automatically according to student’s performance in spelling vocabulary. The vocabulary difficulty is defined according to its length.

**Fig. 8:**
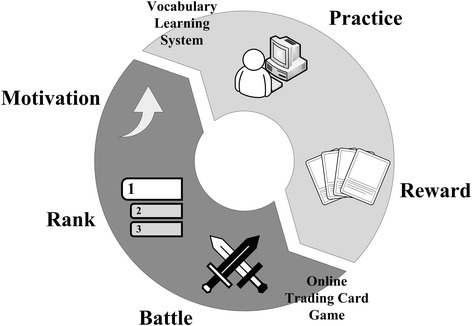
Motivation enhancement cycle

The connections between the learning and the reward make students want to use the web-based learning system and to challenge the spelling quizzes in order to get more valuable cards. Figure 8 shows the motivation enhancement cycle that this research proposes. Firstly, students use the learning system to practice their English spelling. Then, the learning system automatically gives corresponding cards of the game as rewards based on their performance. After students collect enough cards to play the game, they can sign-in the game to fight with their fellows. For those students who do not want to compete with others, they may have interest in either watching their classmates’ matches or simply collecting the cards. The cards are collectable just like coins, stamps, and hockey cards; students may want to see they have all higher-level cards as well as rarer cards in their card collection book.

### Hypotheses

**Fig. 9:**
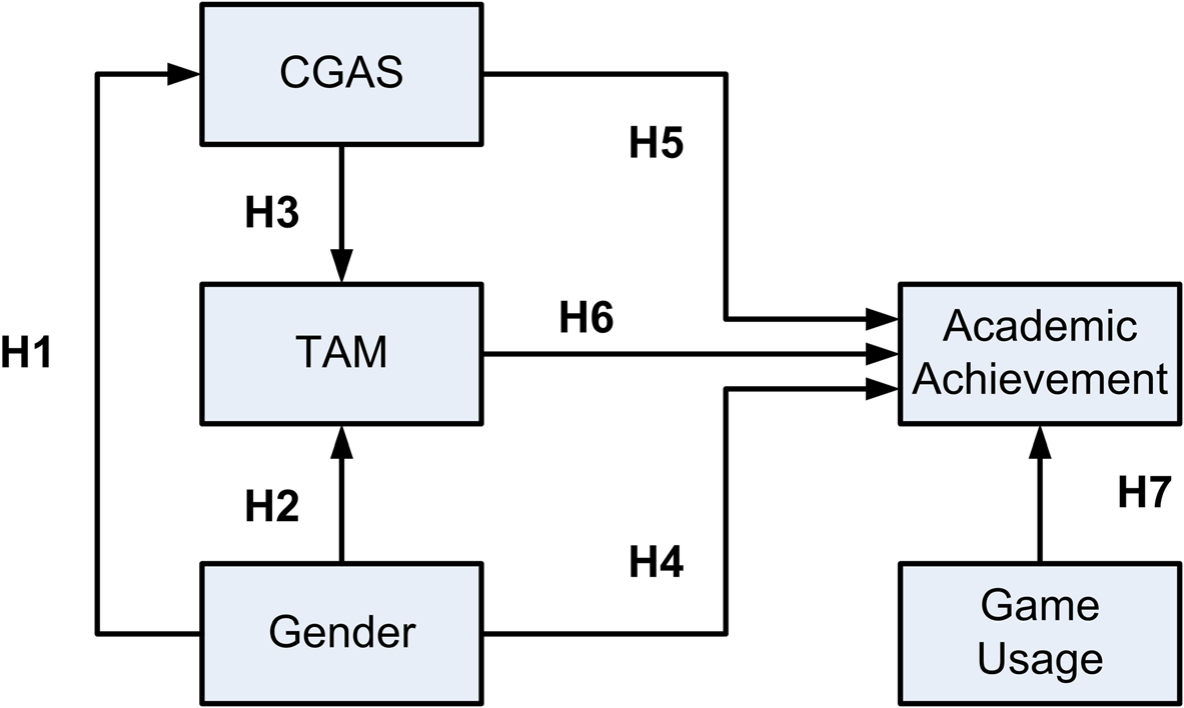
Relations among factors in the hypotheses

There are several hypotheses for using the in-game cards as educational rewards which is illustrated in Fig. 9:

- H1: Students’ gender will affect their attitudes toward computer games.
- H2: Students’ gender will affect their acceptance of having in-game cards as educational rewards.
- H3: Students’ attitudes toward computers will affect their acceptance of having in-game cards as educational rewards.
- H4: Students’ gender will affect their academic achievement.
- H5: Students’ attitudes toward computer games will affect their academic achievement.
- H6: Students’ acceptance of having in-game cards as educational rewards will affect their academic achievement.
- H7: Students’ game usage will affect their academic achievement.

### Pre-test, post-test, and questionnaires

To understand students’ attitudes toward computer games, this research has designed the Computer Game Attitude Scale (CGAS) which was adapted from Chappell and Taylor’s research in 1997 and Liu and colleagues’ research in 2013. Chappell and Taylor first use the term of Computer Game Attitude Scale and have determined that there are three related factors: Anxiety, Liking, and Confidence. In 2013, Liu and colleagues have extended the items developed by Chappell and introduced three sub-scales, which are Cognition, Affection, and Behavior. Learning is the new factor added in the Cognition sub-scale with the Confidence factor; the Liking factor belongs to the Affection sub-scale, and the Behavior sub-scale has two new factors, Participation and Leisure (Liu et al. 2013). The CGAS questionnaire revised in this research only select items from the four factors in the previous CGAS research, which are:

- Confidence: measures students’ confidence of playing computer games.
- Liking: measures degree of how much students like to play the computer games.
- Learning: measures students’ perceptions toward having positive impact on learning via playing computer games.
- Behavior: measures students’ specific behavior in using computer games.

The revised CGAS questionnaire has eighteen 5-point Likert scale items (5 for “Strongly Agree” to 1 for “Strongly disagree”), including five items in the Confidence factor, six items in the Liking factor, four items in the Learning factor, and three items in the Behavior factor. The items are listed in Table 3.

We also adopted the Technology Acceptance Model (TAM) to evaluate students’ acceptance of playing the game and having in-game cards as educational rewards. The Technology Acceptance Model was first introduced by Davis and colleges in 1989 (Davis, Bagozzi, & Warshaw, 1989; Venkatesh, Morris, Davis, & Davis, 2003). This research took and revised only the items of Behavioral Intention to evaluate students’ intention of playing the game and having the in-game cards as reward after the experiment. Table 4 lists the ten 5-point Likert scale items (same as the CGAS questionnaire, 5 for “Strongly Agree” to 1 for “Strongly disagree”).

### The experiment

The research team conducted a pilot experiment in English course held at an elementary school in Taoyuan County, Taiwan. Five classes with 172 fifth-grade students (80 boys and 92 girls) were recruited in the experiment. All students started to learn English since they were in the third grade according to the government’s policy. Two of the classes (class #1 and class #4) were randomly selected as control group which has 68 students with 31 boys and 37 girls. The other three classes (class #2, class #3, and class #5) were assigned as experiment group with 104 students (49 boys and 55 girls). All of the five classes were taught by the same teacher.

**Fig. 10:**
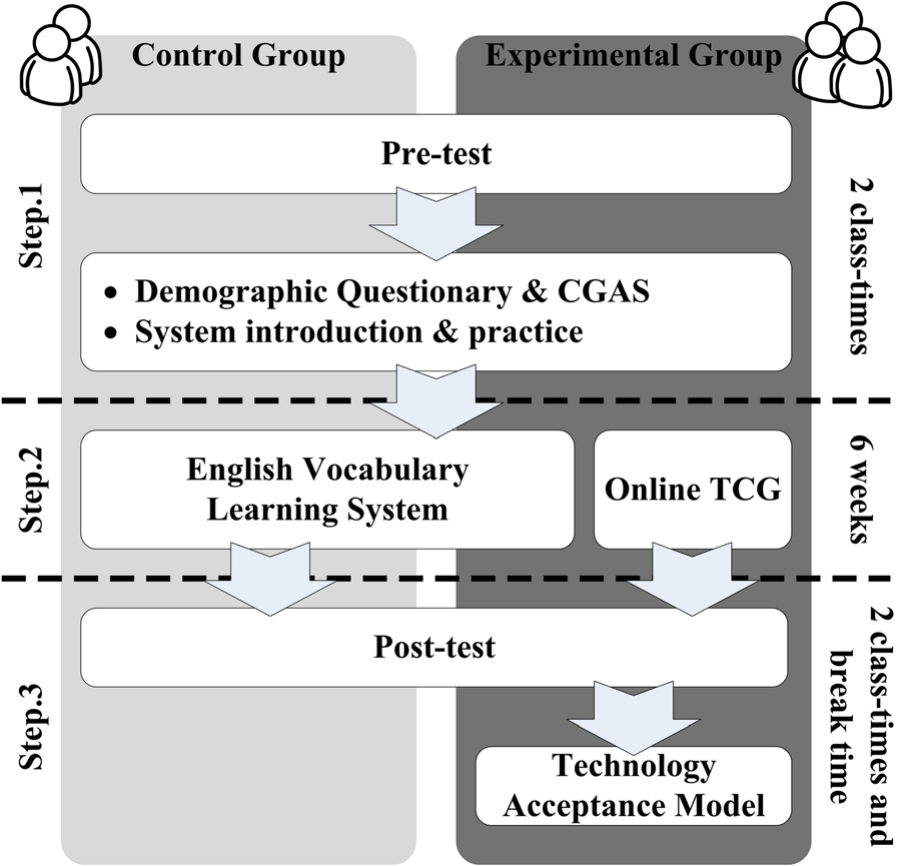
Experiment flow

This experiment included pre-test and post-test which test students’ English vocabulary ability before and after the intervention in order to determine students’ academic achievements. As step 1 in Fig. 10 shows, the students in both groups took pre-test in the beginning and were asked to complete demographic and CGAS questionnaire. After the responses were collected, the research team demonstrated the system(s) involved in the experiment for the students. The control group students were only introduced to the English vocabulary learning system and practiced how to use the system in the class. On the other hand, the research team introduced not only the English vocabulary learning system but also the trading card game for the experiment group students; they had time to practice how to compete with their classmates in the game during the demonstration session in the classes.

In the followed 6 weeks, it is step 2 in Fig. 10. The students in both groups took English vocabulary learning activities and exercises in the learning system without the teacher’s watching. In this period of time, the experiment group students received different levels and rarity in-game cards as rewards according to their performance in the learning system; they can also play the game with others when they have free time after school. At the last step of the experiment, the teacher asked the students to take post-test that covers vocabulary which had been taught in the last 6 weeks. After the test, the experiment group students were asked to complete a Behavioral Intention factor only revised TAM questionnaire for collecting their intention of using the game.

### Reliability and validity

After collecting the data, the research team found out that not all students in the experiment took both pre-test and post-test and some students did not complete the questionnaires; therefore, we removed those students’ entries and had 129 students’ records remained, including those of 50 students (18 boys and 32 girls) from the control group and those of 79 students (36 boys and 43 girls) from the experiment group. Table 2 shows the descriptive statistics data of the students’ demography. Most of the students have computers at their home.

**Table 2:** Descriptive statistics of students’ demography

	Gender		Have computer at home		
	Boy	Girl	No	Yes	No response
Control group	18	32	5	41	4
Experiment group	36	43	3	75	1
Total	54	75	8	116	5

In the next step, the research team used SPSS 17.0 to verify both the validity and reliability for the revised CGAS and TAM questionnaires. Regarding the reliability of the revised CGAS, Cronbach’s alpha value is 0.914, which sits on an “excellent” range and shows the questionnaire is reliable (George and Mallery 2010). The principle component analysis was used to test the validity of the revised CGAS, and the analysis result is valid as Table 3 shows.

**Table 3:** Validity analysis results of the revised Computer Game Attitude Scale

Item	Factor			
	1	2	3	4
Factor 1: confidence				
A12: I am the guy who understands and plays computer games well.	0.871			
A13: I am a skilled computer game player.	0.838			
A11: Playing computer games is easy to me.	0.818			
A10: I am good in playing computer games.	0.786			
A14: After installing computers, I can also install and setup other required software for specific computer game.	0.540			
Factor 2: liking				
A2: I feel comfortable while playing computer games.		0.750		
A6: I am in good mood while playing computer games.		0.730		
A8: I usually play computer games after I finish a course exam.		0.683		
A7: Playing computer games is part of my life.		0.633		
A1: I agree with the instructor to use computer games as part of the course.		0.602		
A9: When I have free time, I play computer games.		0.594		
Factor 3: Learning				
A17: Playing computer games enhances my imagination.			0.814	
A16: Playing computer games makes me have better coordination on eyes and hands.			0.713	
A18: Playing computer game improves my typing skill.			0.666	
A15: I get more involved in the course after I play the educational game.			0.520	
Factor 4: Behavior				
A4: I keep the question in my mind if I have a pending quest/question/mission in the computer game.				0.811
A3: I am very interested in solving quests/questions/missions in computer games.				0.675
A5: I keep finding the solution if I have a quest/question/mission which I cannot solve in the computer game.				0.663
Eigenvalue	7.572	1.918	1.355	1.100
% of variance	42.065	10.655	7.528	6.112
Overall α = 0.914, total variance explained is 66.360%				

The Cronbach’s alpha value of the reliability test for the Behavioral Intention factor only revised TAM is 0.925 represents the questionnaire is also excellently reliable. Table 4 shows the principle component analysis results which present the revised TAM’s validity.

**Table 4:** Validity analysis results of the Behavioral Intention factor only revised TAM questionnaire

Item	Factor 1
Factor 1: behavioral intention	
B7: I would like to use systems similar to the TCG.	0.893
B6: I would like to introduce the TCG to others to play the game.	0.887
B8: If my friends are playing the TCG, I also like to play.	0.866
B5: I would like to keep playing the TCG.	0.856
B3: I would like to play the TCG even the teacher didn’t ask me to play it.	0.797
B9: If I see other players are in the match, I would also like to join the match.	0.790
B2: I would like to play the TCG even I am at home.	0.787
B1: After playing the TCG, I would like to collect cards in the game.	0.681
B4: I would like to get cards in the TCG as rewards from teachers	0.637
B10: If I see other players are in the match, I would like to watch the match.	0.592
Eigenvalue	6.166
% of variance	61.658
Overall α = 0.925, total variance explained is 61.658%	

## Results and discussions

The research investigated students’ experiences in terms of playing video games, handheld games, and computer games. There are 89.9% of students (116 of 129) have played computer game(s) and fewer students have played video game(s) (70.5%) and handheld game(s) (51.2%) as Table 5 lists. The chi-square test shows that there is no significant difference between the control and experiment group on students’ gaming experience (video game *χ* ^2^(1, *n* = 129) = 0.026, *p* = 0.514 > 0.05; handheld game *χ* ^2^(1, *n* = 129) = 1.361, *p* = 0.162 > 0.05; computer game *χ* ^2^(1, *n* = 129) = 0.025, *p* = 0.573 > 0.05).

**Table 5:** Descriptive statistics of students’ game experience

	Have played video game(s)			Have played handheld game(s)			Have played computer game(s)		
	No	Yes	No response	No	Yes	No response	No	Yes	No response
Control group	14	34	2	21	29	0	4	45	1
Experiment group	22	57	0	41	37	1	7	71	1
Total	36	91	2	62	66	1	11	116	2

Students’ CGAS scores in both experiment and control groups also show no significant difference. Table 6 shows the analysis results of the independent *t* test on students’ CGAS scores. The students in both groups have high score in the Liking factor, showing that most of them like to play computer games. The other three factors in the revised CGAS also show high scores, indicating that the students in both groups have positive attitudes toward computer games.

**Table 6:** Independent *t* test result for students’ CGAS score in control and experiment groups

		Descriptive statistic			*t* test		
		*N*	Mean	SD	*t*	*df*	*p*
Confidence	Control	50	3.83	1.193	0.519	127	0.605
	Experiment	79	3.71	1.318			
Liking	Control	50	4.23	0.924	−0.519	127	0.605
	Experiment	79	4.32	0.932			
Learning	Control	50	3.85	1.023	−0.687	127	0.494
	Experiment	79	3.98	1.076			
Behavior	Control	50	4.20	1.004	1.375	127	0.172
	Experiment	79	3.92	1.244			

The research team also used *t* test to verify hypothesis H1 to understand whether or not gender will affect students’ attitude toward computer games; if there is gender difference in game attitude, there might also be gender difference on students’ intention of using in-game cards as educational rewards. The results listed in Table 7 show that there are significant differences in all four CGAS factors; scores given by boys are higher than those given by girls. For both of boys and girls, the highest average score given is in the Liking. However, the lowest average score factors are gender different. Girls give lowest score in the Confidence, but boys give lowest in the Learning. The results show that girls have lower confidence of playing computer games; on the other hand, boys do not really think computer games could help as learning tools.

**Table 7:** Independent *t* test result for students’ CGAS score (gender difference analysis)

		Descriptive statistic			*t* test		
		N	Mean	SD	*t*	*df*	*p*
Confidence	Boy	54	4.28	0.993	4.423	126.682	0.000*
	Girl	75	3.38	1.315			
Liking	Boy	54	4.53	0.675	2.786	125.781	0.006*
	Girl	75	4.11	1.041			
Learning	Boy	54	4.16	0.975	2.105	127	0.037*
	Girl	75	3.77	1.084			
Behavior	Boy	54	4.47	0.797	4.152	124.722	0.000*
	Girl	75	3.71	1.278			

*: *p* < 0.05

Because there is gender difference in students’ attitudes toward games, we are not surprised to see that there is also significant difference between boys and girls in their Behavioral Intention of using the game (hypothesis H2) which was verified by the independent *t* test. As Table 8 shows, boys’ Behavioral Intention scores are higher than girls’ in all items. The scores in some items also have significant gender difference. Boys have higher intention of collecting in-game cards as educational rewards (B1 and B4) and want to continuously play the game after the experiment (B5), especially when their friends are playing the game (B8) or when they are seeing other players playing the game (B9). They also have higher intention of introducing the game to their friends (B6) and playing other games similar to the proposed game (B7).

**Table 8:** Independent *t* test result for students’ Behavioral Intention score (gender difference analysis)

		Descriptive statistic			*t* test		
		N	Mean	SD	*t*	*df*	*p*
B1: After playing Online TCG, I would like to collect cards in the game.	Boy	36	4.22	1.124	2.080	76.812	0.041*
	Girl	43	3.63	1.415			
B2: I would like to play Online TCG even I am at home.	Boy	36	4.03	1.183	1.072	77	0.287
	Girl	43	3.72	1.333			
B3: I would like to play Online TCG even the teacher didn’t ask me to play it.	Boy	36	4.03	1.230	1.193	77	0.237
	Girl	43	3.67	1.375			
B4: I would like to get cards in Online TCG as rewards from teachers	Boy	36	4.14	1.437	2.805	77	0.006*
	Girl	43	3.14	1.684			
B5: I would like to keep playing Online TCG.	Boy	36	4.31	1.142	2.184	76.864	0.032*
	Girl	43	3.67	1.426			
B6: I would like to introduce Online TCG to others to play the game.	Boy	36	4.22	1.072	2.479	75.669	0.015*
	Girl	43	3.51	1.470			
B7: I would like to use systems similar to Online TCG.	Boy	36	4.33	0.986	3.000	74.305	0.004*
	Girl	43	3.51	1.437			
B8: If my friends are playing Online TCG, I also like to play.	Boy	36	4.36	0.961	2.505	73.201	0.014*
	Girl	43	3.67	1.459			
B9: If I see other players are in the match, I would also like to join the match.	Boy	36	4.50	0.941	3.754	74.039	0.000*
	Girl	43	3.51	1.384			
B10: If I see other players are in the match, I would like to watch the match.	Boy	36	4.03	1.320	1.752	77	0.084
	Girl	43	3.47	1.502			
Average	Boy	36	4.22	0.817	3.020	75.397	0.003*
	Girl	43	3.55	1.136			

*: *p* < 0.05

To investigate the relations between CGAS and TAM (hypothesis H3), the research team uses Pearson correlation to find out the linear dependence between these two factors. The analysis results are listed in Table 9, revealing the relations between students’ intention of using the game (and the in-game cards as rewards) and their attitudes toward computer games are positively significantly related.

**Table 9:** Correlation analysis between students’ Behavioral Intention and attitudes toward computer games

	Confidence	Liking	Learning	Behavior
Pearson correlation	0.344	0.468	0.323	0.344
Sig.	0.002*	0.000*	0.004*	0.002*
*N*	79	79	79	79

*: *p* < 0.05

Regarding the verification of hypothesis H4, this research used *t* test to examine whether or not there is a significant difference between boys’ and girls’ academic achievements. The result is listed in Table 10. Both pre-test and post-test have no significant gender difference.

**Table 10:** Independent *t* test result for students’ academic achievements (gender difference analysis)

		Descriptive statistic			*t* test		
		N	Mean	SD	*t*	*df*	*p*
Pre-test	Boy	54	56.78	32.465	−0.357	127	0.721
	Girl	75	58.77	30.399			
Post-test	Boy	54	56.52	27.705	0.172	127	0.864
	Girl	75	55.73	23.868			

To further look at possible gender difference in academic achievements, the research team also does *t* test in both the experiment group and control group and the results are listed in Table 11. Boys and girls in both groups have no significant difference in pre-test and post-test. Therefore, we can say that students’ academic achievements in terms of English vocabulary learning will not be affected by gender. Hypothesis H4 is rejected.

**Table 11:** Independent *t* test result for students’ academic achievements in the experiment and control groups (gender difference analysis)

			Descriptive statistic			*t* test		
			N	Mean	SD	*t*	*df*	*p*
Experiment group	Pre-test	Boy	36	45.72	27.519	−0.939	77	0.351
		Girl	43	51.63	28.116			
	Post-test	Boy	36	51.50	28.415	−0.797	77	0.428
		Girl	43	56.33	25.373			
Control group	Pre-test	Boy	18	78.89	30.826	1.150	48	0.256
		Girl	32	68.38	31127			
	Post-test	Boy	18	66.56	23.900	1.735	48	0.086
		Girl	32	54.94	22.056			

Students’ computer game attitudes also have no significant influence to their academic achievements (hypothesis H5) as Table 12 shows. Although the relation between pre-test and Liking has significant relation, the correlation *r* is −0.189, which shows the two factors only have very weak linear relationship. The result indicates that students’ academic achievement will not be affected by their attitudes toward computer games. Table 12 also lists the results of the correlation analysis between students’ Behavioral Intention and their academic achievements. None of the academic achievement has significant relation to the Behavioral Intention factor, pointing out that students’ intention of using the game (or having the in-game cards as rewards) will not be affected by their academic achievements.

**Table 12:** Correlation analysis between students’ academic achievements and CGAS factors as well as Behavioral Intention

		CGAS				Behavioral intention
		Confidence	Liking	Learning	Behavior	
Pre-test	Pearson correlation	−0.170	−0.189	−0.103	−0.116	−0.131
	Sig.	0.054	*0.032*	0.245	0.189	0.251
	*N*	129	129	129	129	79
Post-test	Pearson correlation	−0.146	−0.121	−0.081	−0.085	−0.142
	Sig.	0.099	0.171	0.359	0.336	0.211
	*N*	129	129	129	129	79
Difference (post-test-pre-test)	Pearson correlation	0.092	0.163	0.066	0.085	−0.018
	Sig.	0.301	0.065	0.457	0.341	0.872
	N	129	129	129	129	79

To understand whether or not using in-game cards as rewards can improve students’ academic achievements, the research team first uses *t* test to examine whether or not the control group students in the two classes have similar level for prior knowledge. The result listed in Table 13 shows that there is significant difference between class #1 and class #4.

**Table 13:** Independent *t* test result for students’ academic achievements in different groups

		Descriptive statistic			*t* test		
		*N*	Mean	SD	*t*	*df*	*p*
Pre-test	Class #1	18	48.78	29.138	−4.533	29.912	0.000*
	Class #4	32	85.31	23.863			

*: *p* < 0.05

On the contrary, when the research team uses ANOVA to evaluate whether or not the experiment group students in different classes performed significantly different in pre-test, the result listed in Table 14 shows students in the three classes have similar prior knowledge level before the experiment. The research team interviewed the teacher regarding the possible reason that students in the two classes have significant difference in their pre-test performance; however, the teacher also had no clue and could not answer it. One possibility we thought is that parents in Taiwan sometimes would send their children to the supplementary learning classes after the school and perhaps more students in the particular class in the control group were the case. On the other hand, since the lecture content the teacher taught in the school may not be identical with that the instructors in supplementary learning classes taught, those students will not benefit from the classes, and their post-test performances reflect what they really learned in the school as well as with the web-based English vocabulary learning system.

**Table 14:** Independent *t* test result for students’ academic achievements in different groups

		Descriptive statistic			ANOVA		
		*N*	Mean	SD	*F*	*df*	*p*
Pre-test	Class #2	26	46.77	31.046	0.276	(2, 76)	0.759
	Class #3	27	47.85	26.804			
	Class #5	26	52.23	26.213			

In the next step, the research team uses *t* test to examine the students’ pre-test and post-test and the difference between the pre-test and post-test between the two groups. As Table 15 shows, the students in the two groups have significant difference in pre-test; students in the control group actually have better performance for pre-test than those in the experiment group. After the 6-week study, the difference between the two groups disappeared; the average score in the experiment group is increased, but the score in the control group is decreased; thus, the two groups show significant difference from pre-test to post-test.

**Table 15:** Independent *t* test result for students’ academic achievements in different groups

		Descriptive statistic			*t* test		
		*N*	Mean	SD	*t*	*df*	*p*
Pre-test	Control	50	72.16	31.123	4.410	127	0.000*
	Experiment	79	48.94	27.825			
Post-test	Control	50	59.12	23.187	1.087	127	0.279
	Experiment	79	54.13	26.736			
Difference (post-test-pre-test)	Control	50	−13.04	20.671	−5.858	62.225	0.000*
	Experiment	79	5.19	9.842			

^*^: *p* < 0.05

Because students in class #4 have better prior knowledge, the research team removes the data of class #4 from the control group and does the *t* test again to compare only class #1 and the experiment group students’ performance in pre-test and post-test and the difference from pre-test to post-test. The result is listed in Table 16. Although the students in class #1 and the experiment group have no significant difference in their performance on both pre-test and post-test, the average score that the experiment group students has is increased and the average score that the class #1 students has is decreased; therefore, the *t* test result shows that the students’ academic achievement changes in class #1 and the experiment group have significant difference.

**Table 16:** Independent *t* test result for students’ academic achievements between class #1 and the experiment group

		Descriptive statistic			*t* test		
		*N*	Mean	SD	*t*	*df*	*p*
Pre-test	Class #1	18	48.78	29.138	0.022	95	0.983
	Experiment	79	48.94	27.825			
Post-test	Class #1	18	46.44	24.288	−0.909	95	0.266
	Experiment	79	54.13	26.736			
Difference (Post-test-pre-test)	Class #1	18	−2.33	12.728	−2.988	95	0.006*
	Experiment	79	5.19	9.842			

*: *p* < 0.05

There are two supplemental questions asking students’ perceived ease of use toward the game. Although there are significant differences between boys’ and girls’ responses as Table 17 lists, all of them respond positively to the first question, “The user interface of Online TCG is easy to use.” Girls perceived a little bit less positive to the second question, “It is easy to learn how to use Online TCG,” showing that girls might have more difficulty in learning how to play a new game.

**Table 17:** Independent *t* test result for students’ perceived ease of use of the game (gender difference analysis)

		Descriptive statistic			*t* test		
		*N*	Mean	SD	*t*	*df*	*p*
C1: The user interface of Online TCG is easy to use	Boy	36	4.56	0.735	2.320	71.112	0.023*
	Girl	43	4.05	1.194			
C2: It is easy to learn how to use Online TCG.	Boy	36	4.67	0.717	3.642	68.181	0.001*
	Girl	43	3.84	1.271			

*: *p* < 0.05

## Findings and recommendation

### Findings from the experiment results

Based on the experiment results, we have the following two findings:

1. Boys have higher intention of using in-game cards as educational rewards
  Although the analysis result listed in Table 8 indicates that boys have higher intention of using the game and having the in-game cards as rewards, many other researchers believe that females have higher intention to play educational game on the contrary (Hwang et al. 2013; Lu et al. 2011). There are two possible reasons that boys responded more positively to Behavior Intention factor in this research:
  Boys prefer playing competitive games (Hartmann and Klimmt 2006), such as first-person shooter videogame (Jung et al. 2014). The proposed game is a kind of competitive game and is easier to engage male players.
  Girls have higher competition anxiety than boys (Hwang et al. 2013; Chen 2010) and are less risk-taking in gaming than their counterpart (Szell and Thurner 2013); social games might be much easier to engage girls (Manero et al. 2016).
  However, some researchers believe that females have higher acceptance to the game-based learning systems because females have higher perceived usefulness toward educational games (Hwang et al. 2013); male students might have lower intention of using educational game for learning. If male students enjoy playing the game and have higher intention of getting in-game cards as rewards of learning activities, they might have better academic performance.
2. Students who have more positive attitudes toward computer games have higher intention of using the game and having the in-game items as rewards
  Many studies show that students who spend more time on playing games have lower academic achievements (Brunborg et al. 2014). How to encourage students who have game addition to focus on learning is an important issue. Based on the result listed in Table 9, students who have more positive attitudes toward computer games have higher intention of using the game and having the in-game items as rewards. If students who have lower academic achievement are interested in playing the game, they might want to spend more time and efforts on learning activities to get the rewards. In this case, their academic achievement might be improved.
3. Using the in-game cards as educational rewards can help to improve students’ academic achievements
  Students in the experiment group explicitly say that they want to use the game and have the in-game items as educational rewards (boys’ and girls’ mean values of responses toward Behavior Intention factor are 4.21 and 3.57 as Table 8 lists). The analysis result listed in Table 12 also shows that no matter students have less or higher academic achievements, there is no difference in their behavioral intention of using the game and having the in-game cards as rewards. However, as the results listed in Tables 15 and 16 show, all students’ academic achievements get better as we can tell from the *t* test analysis and the students’ achievement difference (from pre-test to post-test) between the control and experiment groups do show significantly different. These results indicate that the use of the in-game cards as educational rewards can engage students in doing learning activities, no matter what original academic achievements they had earlier before the intervention; overall speaking, students’ academic achievements are improved because of the use of the in-game cards as rewards.

### The recommended process of adopting the proposed TCG as educational reward system

Before a course starts, the teacher can first create accounts for her or his students (or enroll students with their existing accounts created or self-registered earlier). All students will receive a beginner’s card set automatically, and they can start to play the game by organizing the cards into different decks based on their preferred game-play strategies. The teacher can start to think of when is good timing to give students rewards (e.g., quizzes and inquiries in the class, assignments, and exams) and what cards would be better to reflect the students’ performance and could be rewards (e.g., different card types and rarity).

When the course starts, students are going to do many different learning activities in the class, out of school as well as at home, for instances, discussions and question and answering in the class, doing group projects and worksheets in field trip, and writing assignments at home. The teacher can give students different types of cards according to the types of learning activities such as giving student magic cards for assignments, trap cards for discussions, and avatar cards for exams and quizzes. Moreover, the better the performance the students reach, the rarer and higher-level cards the teacher can give to them as rewards.

During the semester, students can play the game with their peers at any time as long as they have a computer which has Internet available and are allowed by their parents, teachers, and school. When they play the game, they can freely use all the awarded cards and the rarer and higher-level cards they have which might make them defeat their opponents easier. Also, since the game allows two players to fight with each other and also two players watch the match as audience, the game’s multiplayer feature can attract students to play, watch, and watch-only the game. Students may also want to observe their future opponents’ strategy beforehand or learn from high-ranking players. For students who neither like to play the game nor watch the game, they might want to collect more and more cards to make their card collection book complete.

In either player or non-player case, the students would be engaged to perform better in the learning activities, because players who lose their matches may want to get better, rare, and higher-level cards to win the game more often; players who win their matches may want to have more powerful cards to keep winning and keep their positions on the ranking list; and non-players may want to make their card collection book complete by collecting different cards from different learning activities. It would be very important for the teacher to plan ahead before the course starts and ensure that students can get different cards as rewards from different learning activities so they can be continuously motivated. If multiple courses in a school are adopting the trading card game, then the effect of the game-based educational reward mechanism will be kept in student’s mind and the learning motivation engaged by the mechanism can be carried to the followed course.

## Conclusion

This research describes an in-house trading card game which can be adopted by teachers and played freely by students. Teachers can give students in-game cards that can be used by students while playing with others as rewards of their performances on different designated learning activities. Also, since the game can be accessed by anyone in the world and supports multiple languages, students might get more motivated since they can play the game with others who may come from different classes, grades, and schools and even different countries and continents.

An experiment is done to evaluate the effectiveness of the using of cards in trading card game as education rewards. The important findings are:

1. Girls are less confident on playing computer games.
2. Boys have higher intention of playing the game and having the in-game items as educational rewards.
3. Students who have more positive attitudes toward computer games have higher intention of using the game and having the in-game items as educational rewards.
4. Using the in-game cards as educational rewards can help to improve students’ academic achievements no matter they are boys or girls, are high-achievement or low-achievement students, and have high intention or low intention of using the game and having the in-game items as education rewards.

In the future work, the research will discuss how to help teachers deliver and give in-game cards automatically in the learning management system (e.g., Moodle) based on students’ learning performance on different learning activities. On the other hand, boys are more engaged in this study; how to integrate other mechanisms, such as achievements without competitive factors, to attract girls to accept in-game cards as educational rewards could be an important issue. Moreover, students might feel bored after they have collected most of the cards or have played the game for a long period. Updating the game with adding new card types might attract students who prefer collecting cards attention again. The teacher could also hold a TCG tournament which might also encourage students collecting more powerful cards for competing with opponents in the tournament.

## Footnotes

1
http://tcg.is-very-good.org

## References

[CR1] Apperley TH (2006). Genre and game studies: toward a critical approach to video game genres. Simulation and Gaming.

[CR2] Barzilai S, Blau I (2014). Scaffolding game-based learning: impact on learning achievements, perceived learning, and game experiences. Computers & Education.

[CR3] Boyle T (1997). Design for multimedia learning.

[CR4] Brunborg GS, Mentzoni RA, Froyland LR (2014). Is video gaming, or video game addition, associated with depression, academic achievement, heavy episodic drinking, or conduct problems?. Journal Behavioral Additions.

[CR5] Chappell KK, Taylor CS (1997). Evidence for the reliability and factorial validity of the computer game attitude scale. Journal of Educational Computing Research.

[CR6] Chang C, Chang M, Heh J-S, Kinshuk, Huang R (2014). National palace museum adventure—a mobile educational role playing game for museum learning. Ubiquitous learning environments and technologies.

[CR7] Chang, C, Wu, S, Chang, M, & Heh, J.-S (2008). Activity generator for informal learning in museum. In *Proceedings of the 7th WSEAS International Conference on E-ACTIVITIES (E-ACTIVITIES 2008)* (pp. 189–194).

[CR8] Chen L (2010). The impact of perceived risk, intangibility and consumer characteristics on online game playing. Computers in Human Behavior.

[CR9] Chen P, Kuo R, Chang M, Heh J-H (2009). Designing a trading card game as educational reward system to improve students’ learning motivations. Transactions on Edutainment, III.

[CR10] Cheville RA (2016). Linking capabilities to functionings: adapting narrative forms from role-playing games to education. Higher Education.

[CR11] Dankbaar MEW, Alsma J, Jansen EEH, van Merrienboer JJG, van Saase JLCM, Schuit SCE (2016). An experimental study on the effects of a simulation game on students’ clinical cognitive skills and motivation. Advances in Health Sciences Education.

[CR12] David-Marshall, B, Dreunen, JV, & Wang, M (2010). Trading card game industry—from the T to the C to the G. SuperData Research, Inc., New York.

[CR13] Davis FD, Bagozzi RP, Warshaw PR (1989). User acceptance of computer technology: a comparison of two theoretical models. Management Science.

[CR14] George D, Mallery P (2010). SPSS for windows step by step: a simple guide and reference 18.0 update.

[CR15] Hainey T, Connolly TM, Boyle EA, Wilson A, Razak A (2016). A systematic literature review of game-based learning empirical evidence in primary education. Computers & Education.

[CR16] Hallford, N, & Hallford, J (2001). Swords and circuitry: a designer’s guide to computer role playing games. Roseville, CA: Prime Publishing. Upper Saddle River, NJ: FT Press

[CR17] Hartmann T, Klimmt C (2006). Gender and computer games: exploring females’ dislikes. Journal of Computer-Mediated Communication.

[CR18] Hwang M-Y, Hong J-C, Cheng H-Y, Peng Y-C, Wu N-C (2013). Gender differences in cognitive load and competition anxiety affect 6th grade students’ attitude toward playing and intention to play at a sequential or synchronous game. Computers & Education.

[CR19] Jung Y, Oh HJ, Sng J, Kwon JH, Detenber BH (2014). Revisiting gender preference for a first-person shooter videogame: effects of non-verbal sensitivity and gender on enjoyment. Interacting with Computers Advance Access.

[CR20] Klopfer E, Sheldon J, Perry J, Chen VH (2012). Ubiquitous games for learning (UbiqGames): Weatherlings, a worked example. Journal of Computer Assisted Learning.

[CR21] Kohn A (1999). Punished by rewards: the trouble with gold stars, incentive plans, A’s, praise, and other bribes.

[CR22] Liu EZ-F, Lee C-Y, Chen J-H (2013). Developing a new computer game attitude scale for taiwanese early adolescents. Educational Technology and Society.

[CR23] Lu C, Chang M, Kinshuk D, Huang E, Chen CW (2011). Usability of context-aware mobile educational game. Knowledge Management & E-Learning: An International Journal (KM&EL).

[CR24] Maguth BM, List JS, Wunderle M (2015). Teaching social studies with video games. The Social Studies.

[CR25] Malone TW (1981). Toward a theory of intrinsically motivating instruction. Cognitive Science: A Multidisciplinary Journal.

[CR26] Malone TW, Lepper MR, Snow RR, Farr MJ (1987). Making learning fun: a taxonomy of intrinsic motivations for learning. Aptitude, Learning and Instruction III.

[CR27] Manero B, Terrente J, Freire M, Fernandez-Manjon B (2016). An instrument to build a gamer clustering framework according to gaming preferences and habits. Computers in Human Behavior.

[CR28] McNinch GW (1997). Earning by learning: changing attitudes and habits in reading. Reading Horizons.

[CR29] Pedro L, Santos C, Aresta M, Almeida S (2015). Peer-support badge attribution in a collaborative learning platform: the SAPO Campus case. Computers in Human Behavior.

[CR30] Rideout VJ, Foehr UG, Roberts DF (2010). Generation M2: media in the lives of 8- to 18-year-olds.

[CR32] Schwabe G, Göth C (2005). Mobile learning with a mobile game: design and motivational effects. Journal of Computer Assisted Learning.

[CR33] Sheldon, J, Perry, J, Klopfer, E, Ong, J, Chen, VHH, Tzuo, PW, & Rosenheck, L (2010). Weatherlings: a new approach to student learning using web-based mobile games. In *Proceedings of the Fifth International Conference on the Foundations of Digital Games* (pp. 203–208). ACM.

[CR34] Siwek SE (2014). Video games in the 21th century: the 2014 report.

[CR35] Steinman RA, Blastos MT (2002). A trading‐card game teaching about host defence. Medical Education.

[CR36] Szell M, Thurner S (2013). How women organize social networks different from men. Scientific Reports.

[CR37] Venkatesh V, Morris MG, Davis GB, Davis FD (2003). User acceptance of information technology: toward a unified view. MIS Quarterly.

[CR38] Virvou, M., Katsionis, G., and Manos, K. (2005). Combining Software Games with Education: Evaluation of its Educational Effectiveness. *Educational Technology & Society, 8*(2), 54–65.

[CR39] Wang, H, & Sun, CT. (2011, September). Game reward systems: gaming experiences and social meanings. In *Proceedings of DiGRA 2011 Conference: Think Design Play* (pp. 1–12).

[CR40] Winefield AH, Barnett JA, Tiggemann M (1984). Learned helplessness and IQ differences. Personality and Individual Differences.

[CR41] Witzel BS, Mercer CD (2003). Using rewards to teach students with disabilities. Remedial & Special Education.

[CR42] Wu SC, Elliott RT (2008). A study of reward preference in Taiwanese gifted and nongifted students with differential locus of control. Journal for the Education of the Gifted.

